# Incremental Learning for Online Data Using QR Factorization on Convolutional Neural Networks

**DOI:** 10.3390/s23198117

**Published:** 2023-09-27

**Authors:** Jonghong Kim, WonHee Lee, Sungdae Baek, Jeong-Ho Hong, Minho Lee

**Affiliations:** 1 Department of Neurology, Keimyung University Dongsan Hospital, Keimyung University School of Medicine, Daegu 42601, Republic of Korea; jonghong89@gmail.com (J.K.); harukuma1049@gmail.com (W.L.); neurohong79@gmail.com (J.-H.H.); 2 Department of Medical Informatics, Keimyung University School of Medicine, Daegu 42601, Republic of Korea; 3 Graduate School of Artificial Intelligence, Kyungpook National University, Daegu 41566, Republic of Korea; scar9cube@gmail.com; 4 Biolink Inc., Daegu 42601, Republic of Korea

**Keywords:** image processing, incremental learning, convolutional neural network, deep learning, artificial intelligence, compressed sensing

## Abstract

Catastrophic forgetting, which means a rapid forgetting of learned representations while learning new data/samples, is one of the main problems of deep neural networks. In this paper, we propose a novel incremental learning framework that can address the forgetting problem by learning new incoming data in an online manner. We develop a new incremental learning framework that can learn extra data or new classes with less catastrophic forgetting. We adopt the hippocampal memory process to the deep neural networks by defining the effective maximum of neural activation and its boundary to represent a feature distribution. In addition, we incorporate incremental QR factorization into the deep neural networks to learn new data with both existing labels and new labels with less forgetting. The QR factorization can provide the accurate subspace prior, and incremental QR factorization can reasonably express the collaboration between new data with both existing classes and new class with less forgetting. In our framework, a set of appropriate features (i.e., nodes) provides improved representation for each class. We apply our method to the convolutional neural network (CNN) for learning Cifar-100 and Cifar-10 datasets. The experimental results show that the proposed method efficiently alleviates the stability and plasticity dilemma in the deep neural networks by providing the performance stability of a trained network while effectively learning unseen data and additional new classes.

## 1. Introduction

Recent incremental learning research has focused on class-wise incremental approaches [1,2,3,4,5]. Most class-wise incremental learning is adopting batch-type learning for each class incremental step. Whereas, in the case of humans, incremental learning is performed in an online way for each datum. For instance, whenever a human learns a concept, he or she does not need a bunch of pictures, but only needs to carefully look over an image, and experience grows through time through such a process. Not only in the human-like model, but in many application areas of industry, data are provided in a real-time manner such as streaming applications. However, recent class-wise incremental learning methods cannot solve such problems. For the cases of such long periodic incremental learning, we call it lifelong learning [6]. In more practical situations, incremental learning should be lifelong because, in the entire lifespan, it is hard to avoid meeting totally new input compared to the already trained data. The lifelong learning scenario assumes the case of training a new task for a network that is already fully trained. Solving this kind of task always meets the catastrophic forgetting problem [7,8]. In this case, rather than suppress the existing knowledge to learn the new task, it would be better to apply it. Therefore, in this paper, we are trying to provide a new solution to the online datum-wise incremental learning problem. We tried to reflect and avoid existing knowledge at the same time when adding a new task functionality to the trained network by using QR factorization. Recently, some new tensor subspace models have been proposed for accurate information expression that can support the feasibility of our proposed model [9,10]. If we do not know what the past data were, and what the future input will be, but only have a trained model and a single input datum, the problem of training the model becomes online datum-wise incremental learning.

The incremental learning problem can be divided into two subproblems. The first problem is how we can learn a new concept, i.e., how we can increase the number of output classes. Second is how we can update pre-existing weights of the network incrementally while avoiding catastrophic forgetting [7]. Our model tries to solve those two problems simultaneously.

In neural networks, an incremental learning problem can be regarded as a process of finding the appropriate weights and biases. A weight can be described by its shape and magnitude. Therefore, we propose a novel method for selecting the biases and deriving the magnitude and shape of the weights.

**Figure 1:**
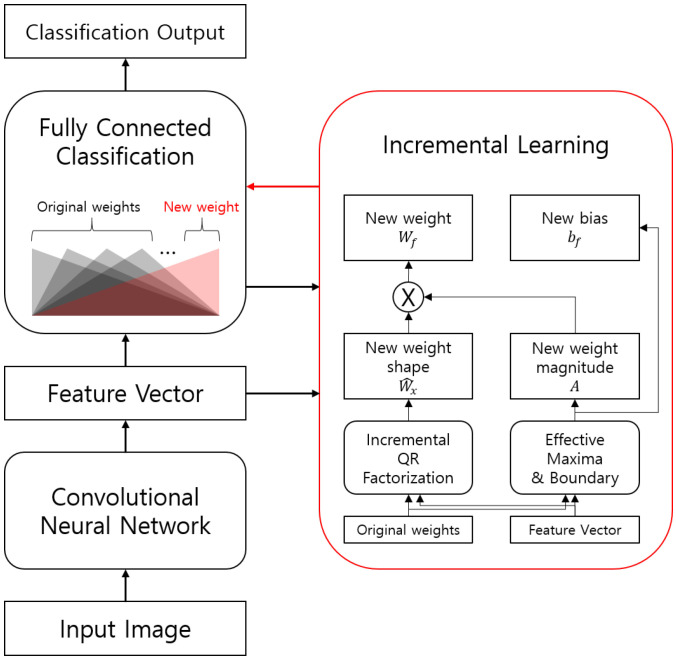
Datum-wise online incremental factorization (DOI) for deep convolutional neural networks. Extracted feature vectors and existing classification weights are used to generate the new weight and bias for the new class using one sample image without backpropagation.

The entire structure of our proposed method is described in Figure 1. In our proposed method, to get the shape of incremental weights, we use QR factorization, which is one of the effective data compressive sensing methods [11]. For the bias selection and magnitude calculation, we apply the new effective maxima and boundary concept. In real-world situations, the activation of a node of a neural network is limited to a maximum value because of the finite origin of an input. In this case, we define the possible maximum outputs as effective maxima and its corresponding input as a center point. For the rectified linear unit (ReLU) activation function [12], there is a zero-crossing point that is regarded as the boundary, and this boundary can be controlled by selecting a bias value. In this way, we can obtain an appropriate weight and bias in an incremental way.

## 2. Related Work

Catastrophic forgetting is an important problem in neural networks [7,8,13,14]. The adaptive resonance theory (ART) [15] network is one of the popular models that was trying to solve the stability–plasticity dilemma [16]. It has advanced to Fuzzy-ART [17] and growing fuzzy topology ART (GFTART) [18]. Only GFTART has class-dependent representation ability using growing fuzzy topology. However, those models are not efficient for real-world applications with complex datasets because of scalability limitations for large sets of data in the real world. Another attempt is copied network approaches [19,20], in which one tries to train a copied network by minimizing the difference between a pre-trained network output and a copied network output for both a pre-trained task and a new task. Therefore, they optimize two different objectives together to avoid the forgetting problem. In addition, there are other approaches that are based on probabilistic implementations [21,22]. Those methods are basically inspired by biological aspects of complementary learning in the human brain. They model a posterior probability of an output layer using the Laplacian approximation and use the Fisher information matrix as a constraint for selective weight update to overcome the catastrophic forgetting problem. Recent incremental learning studies changed the strict online-setting into mild class-wise incremental learning. LwF [2] triggered this trend. Moreover, after iCarl [5], keeping some of the old data is allowed. We can find several derivatives [1,3,4,23,24,25,26] using the exemplar concept of iCarl. Recently, there are exemplar-free models that have been released [27,28]. Compared to the exemplar-based models, those exemplar-free models used variants of the generative networks. However, all of those methods are based on a lot of old and new batch datasets and, therefore, they cannot be regarded as a datum-wise online incremental learning method. As a result, it is not only insufficient for plasticity but also different from the human-like sample-by-sample online incremental learning.

## 3. Methods

We use the pre-trained feature extractor network of VGG16 [29], which is trained with ILSVRC2012 [30]. Therefore, we know that there are already 1000 trained classes. We start incremental learning from this point.

### 3.1. Incremental QR Factorization for Weight Shape Derivation

Let $n_{i}$ denote the number of training images of the *i*th subject in a group of subjects; consequently, when the total number of subjects is *K*, $n=\sum_{i=1}^{K}n_{i}$. The $n_{i}$ column vectors obtained from the *i*th subject comprise a matrix $A_{i}$, and training data matrix *A* is formed as

(1) $$A=[A_{1},A_{1},\cdots ,A_{K}]$$

where

(2) $$A_{i}=\left[a_{i,1},a_{i,2},\cdots ,a_{i,n_{i}}\right]\in R^{m\times n_{i}}$$

where $a_{i,j}$ is the *i*th subject’s *j*th training datum. In [31,32,33], it is assumed that any test image lies in the subspace spanned by the training images belonging to the same class. That is, any test sample *x* can find its associated class without its label information. Further, test sample *x* is reconstructed through a linear combination of existing bases by

(3) x=Aα

Optimal α, which effectively reconstructs the target sample with other training samples, can be obtained in various ways. In the case of the $l_{1}$-minimization algorithm, the n-sparse signal to reconstruct test sample *x*, is computed using

(4) $$\min_{\alpha \in R^{n}}{\left||\alpha |\right|}_{l_{1}}$$

When *m* is large, solving Equation (4) via linear programming becomes computationally too expensive. Wright et al. [32] and Yang et al. [33] used random matrix $\Phi \in R^{d\times m}$ (where d≪m) and computed the vector that minimizes Equation (4), where Φx=ΦAα or Φx−ΦAα≤ε when error tolerance ε>0 is given. Introducing the random matrix $\Phi \in R^{d\times m}$ significantly reduces the computational complexity.

Because of the significant amount of computation incurred, various approaches, such as [34], have been proposed to optimize $l_{1}$-minimization. However, Shi et al. [31] adopted $l_{2}$-minimization instead to exploit its efficiency; $l_{2}$-minimization is defined by

(5) $$\min_{\alpha \in R^{n}}||x-{A\alpha ||}_{l_{2}}^{2}$$

In contrast to $l_{1}$-minimization, $l_{2}$-minimization can be solved using a pseudo-inverse matrix. In addition to this advantage, algorithms using $l_{2}$-minimization can recover test samples more clearly, thereby achieving more accurate performance with less computation.

(6) $$\begin{array}{c} \alpha ={\left(A^{T}A\right)}^{-1}Ax \end{array}$$

(7) $$\begin{array}{c} \alpha =R^{-1}Q^{T}x \end{array}$$

In Equation (5), optimized α can be computed via Equations (6) and (7), which are obtained by solving an equation that equalizes Equation (5)’s derivative with zero. In the result, Equation (5) can be replaced by Equation (6), and the pseudo inverse of *A* is also replaced by the inverse of QR in Equation (7). The inverse matrix of *R* and *Q* can also be used to compute optimal α, after computing it only once in the batch-training phase.

We can reconstruct *x* using *A* and α computed from Equation (7). If the test image *x* is the same as one of the training images, the corresponding α value is activated as 1, and other values go to zero. It is very similar to the one hot vector activation of neural networks. Therefore, we can get the weight shape ${\hat{W}}_{x}$ of corresponding current input sample *x* as follows:

(8) $$R^{-1}Q^{T}=\left[c_{1},c_{2},\cdots ,c_{n},c_{n+1}\right]\in R^{m\times (n+1)}$$

(9) $${\hat{W}}_{x}=c_{n+1}$$

where *m* is the size of the weight, *n* is the number of old output, and $c_{n+1}$ can be regarded as the new incremented weight shape corresponding to input *x*. Here, we finally found the weight shape ${\hat{W}}_{x}$, which will be used to create a new class weight by collaborating with another algorithm from Section 3.3.

The expression *Q* consists of orthogonal and normalized vectors, such as $\left[e_{1},e_{2},\cdots ,e_{n}\right]$, in which each $e_{i}$ is computed using

(10) $$\begin{array}{c} u_{1}=a_{1}\\ u_{2}=a_{2}-proj_{e_{1}}a_{2}\\ u_{3}=a_{2}-proj_{e_{1}}a_{3}-proj_{e_{2}}a_{3}\\ \vdots \\ u_{n}=a_{n}-\sum_{j=1}^{n-1}proj_{e_{j}}a_{n} \end{array}$$

and

(11) $$e_{i}=\frac{u_{i}}{|u_{i}|}$$

where $a_{n}$ is the *n*th training sample vector, and $proj_{e_{j}}a_{n}$ is the projection of $a_{n}$ in the $e_{j}$ direction. In the result of this computation, vectors of *Q* are independent of each other.

*R* consists of coefficient vectors $\left[r_{1},r_{2},\cdots ,r_{n}\right]$, which are for reconstructing the original sample vectors; *R* is computed using

(12) $$R=\left(\begin{array}{cccc} <e_{1},a_{1}> & <e_{1},a_{2}> & <e_{1},a_{3}> & \cdots \\ 0 & <e_{2},a_{2}> & <e_{2},a_{3}> & \cdots \\ 0 & 0 & <e_{3},a_{3}> & \cdots \\ \vdots & \vdots & \vdots & \ddots \end{array}\right)$$

Using Equations (10) to (12), *Q* and *R* matrices are the result of decomposition of *A*, and they possess several important characteristics. First, *Q* is a unitary matrix with the special characteristic $Q^{T}Q=QQ^{T}=I$. This means that the inverse matrix of *Q* is easily computed by transposing *Q*. Second, *R* always has an upper triangular form. The inverse of this upper triangular matrix can be calculated using Gauss–Jordan elimination. Although Gauss–Jordan elimination is not the best for calculating an inverse matrix, it is efficiently applied in the proposed algorithm. Using the above equations, QR decomposition is sequentially computed for each column at once [35,36].

When new training samples are added, these inverse matrices have to be recomputed. From above, we know that *Q* is a unitary matrix, with the inverse of *Q* the same as $Q^{T}$, and *R* is an upper triangular matrix, whose inverse matrix can be calculated in simple iterative fashion via Gauss–Jordan elimination. New data updating can be performed on the basis of these two characteristics. We propose a simple and fast updating method that facilitates incremental learning of new training samples. The matrix *Q* consists of orthonormal bases, as represented in Equations (10) and (11). When new data are available, we can update *Q* and *R* by simply inserting an additional column:

(13) $$Q_{n+1}=\left[Q_{n},e_{n+1}\right]$$

where $e_{n+1}$ is an orthonormalized vector from $a_{n+1}$ by Equations (10) and (11).

Further, because it is a unitary matrix, $Q_{n+1}^{-1}$ can be obtained by transposing $Q_{n+1}$. This means that $Q_{n+1}^{-1}$ can be updated from $Q_{n}^{-1}$ by adding just a row, $e_{n+1}^{T}$; $R_{n+1}^{-1}$ is also updated from $R_{n}^{-1}$ by

(14) $$\begin{array}{c} r_{k,n+1}^{-1}=-\frac{\left(\sum_{i=1}^{n}r_{k,i}^{-1}r_{i,n+1}\right)}{r_{n+1,n+1}} \end{array}$$

(15) $$\begin{array}{c} r_{n+1,n+1}^{-1}=\frac{1}{r_{n+1,n+1}} \end{array}$$

where $r_{i,j}^{-1}$ is matrix $R^{-1}$’s element at the *i*th row and *j*th column. Equations (14) and (15) are derived from Gauss–Jordan elimination, which is commonly used to compute the inverse matrix of the upper triangular matrix. To apply Equations (14) and (15), we need $R_{n+1}$, which consists of $\left[r_{1},r_{2},\cdots ,r_{n},r_{n+1}\right]$; $r_{1},r_{2},\cdots ,r_{n}$ are already known because they were computed in the batch learning phase. Thus, only $r_{n+1}$ is computed in the incremental learning phase. This simple updating method significantly reduces the new data learning time without a big loss of performance. In addition, the incremental QR factorization is to preserve the accurate subspace feature so it is robust to the noisy inputs.

### 3.2. Center and Boundary of Feature Distribution

Our fundamental assumption is that the learning process of a neural network for each node in a hidden layer represents a useful distribution of features according to a specific class. In [21,22], the authors tried to convert the feature distribution to normal distribution using Laplace approximation. The result contains approximation errors during the conversion process. It is difficult to convert specific input with non-Gaussian distribution to a normal distribution. Moreover, a bunch of datasets is needed for a new class for Laplace approximation, which makes it unsuitable for datum-wise online incremental learning in real-world applications. Therefore, we redefine the center and boundary of a node in the last hidden layer using its existing parameters.

A node activation consists of its input, weight, bias, and activation function. In the case of ReLU, which is a biologically plausible activation function [37], the biggest difference between normal distribution and node activation is the dynamic range. Unlike the normal distribution output, which has limited dynamic range, the maximum value of ReLU activation is infinite, ideally. However, in real situations, the ReLU output never reaches an infinite value. There should be a finite maximum, and we define the finite maximum as the effective maximum. The effective maximum has its corresponding input as a weighted sum.

**Figure 2:**
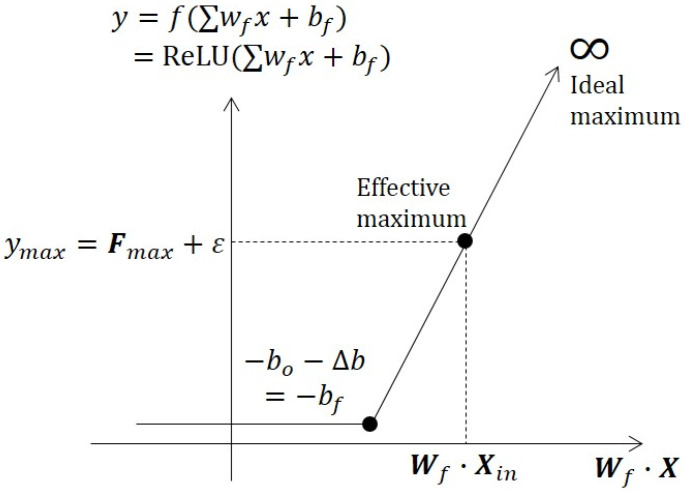
Center and boundary definition in the proposed framework. The center point corresponds to the effective maximum point. In the case of incremental learning, the center point is the input data point. The boundary is defined as the zero crossing point of ReLU. Even if the maximum value of ReLU is ideally infinite, however, there should be a finite effective maximum point in practical situations.

As shown in Figure 2, we describe the ReLU activation function by output *y* axis and input axis of a weighted sum. Therefore, we redefine the center point X with respect to the effective maximum point. The zero crossing point can be described by the negative value of bias. In the case of normal distribution, we can define a receptive field with a threshold point that gives a criterion of the decision boundary. A trained deep neural network has a crisp decision boundary. The decision boundary can be represented by a hyperplane that consists of weight and bias. The weight makes the angle or shape of the hyperplane, and the bias allocates its position. For the ReLU activation, the decision boundary is the zero crossing point of the function. Therefore, we can use the zero crossing point as a decision boundary that is determined by the bias.

### 3.3. Bias Selection and Magnitude Derivation

A pre-trained deep neural network consists of weights and biases in a layered structure. Therefore, even though we infer the existence of the effective maximum, we do not know the exact value and the center point. In the training phase of incremental learning, we have new data. The input can be considered as the center point of its corresponding particular feature. When a pre-trained deep neural network fails to classify new data, then we create a new feature that is more suitable for the new data. The first step of our framework creates a new weight using the current corresponding class weight as the following equation.

(16) $$W_{f}=W_{o}+\Delta W=W_{o}+A{\hat{W}}_{x}$$

where $W_{f}$ is a new weight, $W_{o}$ is a pre-trained weight of the corresponding class of input data, *A* is the scaling factor that decides the weight magnitude, ΔW is a weight update term, and ${\hat{W}}_{x}$ is obtained for the proposed incremental QR factorization (Equation (9)). Since this learning framework should be operated in failure case of classification, the resultant output value using $W_{f}$ should be higher than the wrong classification output value to get a correct result. Therefore, we can infer the following equation to meet the desired condition:

(17) $$y_{f}=W_{f}X_{in}+b_{f}=\left(W_{o}+A{\hat{W}}_{x}\right)X_{in}+b_{o}+\Delta b=F_{max}+\epsilon$$

where $X_{in}$ is input, $b_{o}$ is original input bias, $b_{f}$ is desired bias, and Δb is the update term. To get the correct output, the value of $y_{f}$ should be higher than the highest value of wrong classification results, $F_{max}$. Therefore, we define the target output $y_{f}$ by $F_{max}$ with a small value of ϵ. Finally, we can get the following equation of magnitude factor *A*:

(18) $$A=\frac{F_{max}+\epsilon -y_{o}-\Delta b}{{\hat{W}}_{x}*X_{in}}$$

Note that $y_{o}$ is the original output, which can be calculated by $W_{o}$ and $b_{o}$. The two important parameters are the scaling factor *A* and the bias update factor Δb. If we assume a small value of ϵ, other values are already known. A new decision boundary for new data can be determined by $W_{f}$ and $b_{f}$. The $W_{f}$ and $b_{f}$ depend on *A* and Δb, as shown in Equation (17). Since *A* can be determined by Δb in Equation (18) if we set a correct bias update factor Δb, we can design a new decision boundary for the new data.

For the simplicity of understanding the effect of bias selection, let us assume that $y_{o}=0$. This case is the initial stage of the new class increment. If $\Delta b=F_{max}+\epsilon$, then A=0. Therefore, we can realize that the maximum value of Δb is $F_{max}+\epsilon$, and if we decrease the effect of Δb, then the effect of *A* increases. We suggest the following bias selection equation:

(19) $$\Delta b=r\left(F_{max}+\epsilon \right),\;0<r\leq 1$$

Algorithm 1 gives the pseudo-code for our proposed framework.
**Algorithm 1** Datum-wise online incremental learning.1:$X_{new}$ is new input dataset (or a stream of data)2:*T* is a set of targets with respect to $X_{new}$3:*Y* is a set of classes4:$\Theta_{Y}$ is a set of features5:**for** $i\leftarrow 1\cdots |X_{new}|$ **do**6:   $t_{i}\leftarrow t_{i}\in T$7:   **if** $t_{i}\notin Y$ **then**8:     $W_{o}\leftarrow$ zeros with size of $\theta_{y}$9:     $b_{o}\leftarrow 0$10:     $Y\leftarrow Y\cup \left\{y_{I}\right\}\;where\;y_{i}=t_{i}=ReLU\left(W_{o}x+b_{o}\right)$11:     $\theta_{y_{i}}\leftarrow \left\{W_{o},b_{o}\right\}$12:     $\Theta_{Y}\leftarrow \Theta_{Y}\cup \left\{\theta_{y_{i}}\right\}$13:   **end if**14:   $F_{max}=\underset{y}{argmax}\left\{p(x_{i}|\theta_{y})\right\}\;where\;x_{i}\in X_{new},y\in Y$15:   **if** $F_{max}\neq t_{i}$ **then**16:     $y_{o}\leftarrow p\left(x_{i}|\theta_{t_{i}}\right)$17:     $W_{o},b_{o}\leftarrow \theta_{y_{o}}$18:     $W_{Y}^{\prime }\leftarrow \Theta_{Y}^{\prime }=\left\{\theta_{y}|\theta_{y}\neq \theta_{t_{i}}\right\}$19:     $Q^{\prime },R^{\prime }\leftarrow QR\_factorization\left(W_{Y}^{\prime }\right)$20:     $Q,R\leftarrow Incremental\_QR(Q^{\prime },R^{\prime },x_{i})$21:     ${\hat{W}}_{x}=c_{t_{i}}\leftarrow \left\{c_{1},c_{2},\cdots ,c_{t_{i}}\right\}=R^{-1}Q^{T}$22:     $A=\frac{F_{max}+\epsilon -y_{o}-\Delta b}{{\hat{W}}_{x}*x_{i}}\;where\;\epsilon \ll 1,0\leq \Delta b<F_{max}+\epsilon$23:     $W_{f}=W_{o}+A{\hat{W}}_{x}$24:     $b_{f}=b_{o}+\Delta b$25:     $\theta_{y_{f}}\leftarrow \left\{W_{f},b_{f}\right\}\;where\;y_{f}=ReLU\left(W_{f}x+b_{f}\right)$26:   **end if**27:**end for**

## 4. Experiments and Results

In our experiment, even though the proposed method can be applied to any kind of deep convolutional neural network, we apply our framework on VGGNet 16 layer D model [29] as an example case. The VGGNet consists of 13 convolutional layers and 3 fully connected layers. Our experiment is performed on the output features of the 15th layer and the output weights of the network. We use pre-trained weights of the model for the ILSVRC2012 [30] dataset. The ILSVRC2012 dataset consists of about one million images of 1000 classes with a training time of a few weeks. If we apply new data and/or new classes on this model and re-train the network by the conventional learning algorithm, the catastrophic forgetting problem is very critical. Therefore, with the proposed datum-wise online incremental learning, we will show that our framework overcomes the catastrophic forgetting problem.

The proposed incremental learning includes not only the adaption of new data but also extending the model for additional classes. For the experiments, we use another image dataset, Cifar-100 [38], which includes 100 image classes, 500 images each, and 100 images each for the test. Additionally, we use Cifar-10 [38], which consists of 10 classes, 5000 images each for training, and 1000 images each for testing.

We used the hyperparameters of the proposed framework as ϵ = 0.1 and r = 0.1. The ϵ is selected as about 5% of output value after checking network output. The Δb is selected as the boundary range to be 10% of the existing feature boundary. The performance is measured by top-1 accuracy. We squashed all the data sizes to 224 × 224 to be the same as the input dimension of VGGNet, 4096. All the feature extraction networks are frozen, and only output layers are trained to correspond to our proposed learning method.

### 4.1. Comparison with Class-Wise Incremental Backpropagation

To show how the catastrophic forgetting problem is resolved by our proposed method, we compare it with LwF [2], which is based on backpropagation and distillation learning. We know there are more recent papers, such as iCarl [5] and its derivatives [1,3,4]. Even though those studies give better results, they used old data, which may not be suitable for lifelong incremental learning in real applications. However, since only the LwF does not use old data, we regard the LwF as the most suitable in the comparison. For the LwF setting, we use the learning rate of 0.0001 and warmup for the first epoch. The training process is stopped at each maximum test output. The number of classes for each task is 1, so it is a class-wise increment.

**Figure 3:**
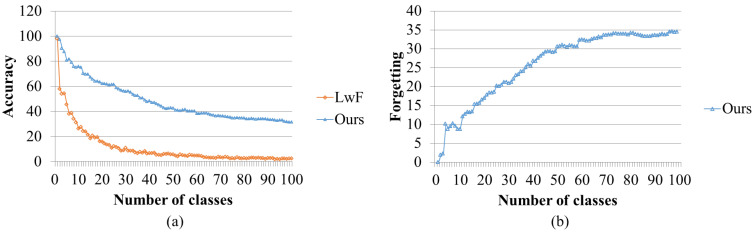
Class-wise incremental learning comparison result on Cifar-100: (**a**) comparison between the proposed model and LwF; (**b**) forgetting the proposed model.

Figure 3 shows that our proposed datum-wise online incremental learning outperforms batch-type class-wise incremental learning with backpropagation. We also visualize forgetting [39] of the proposed model. Final LwF accuracy is 2.4, our proposed model accuracy is 31.48, and forgetting is 34.57.

**Figure 4:**
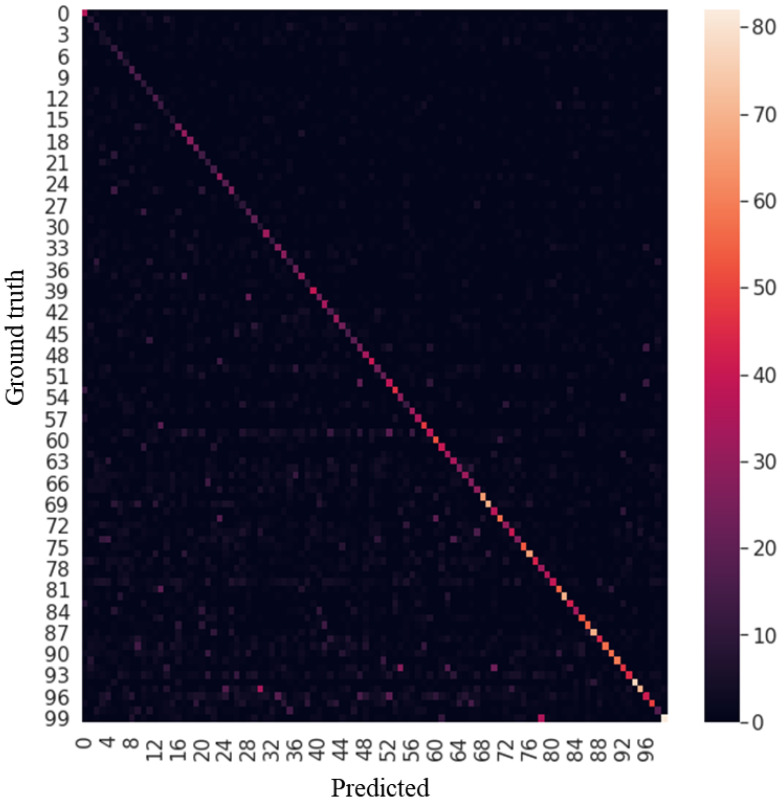
Confusion matrix of class incremental learning on Cifar-100.

Figure 4 shows the confusion matrix of our proposed model. The result indicates that our proposed method performed relatively well at predicting labels, even though a little forgetting happened on old labels compared with recent labels.

### 4.2. Random Input Comparison with One Epoch Backpropagation

For a more realistic experimental setting, let us assume that input data are random. In this case, the proposed model increases the number of classes if it is needed or updates existing classes. This process looks similar to conventional batch learning. The difference is it uses one epoch only in the training process. Therefore, we can compare the performance with one epoch training of the conventional method. For the conventional training, we set the learning rate as 0.01, which gives the best performance, and warmup is also applied.

**Figure 5:**
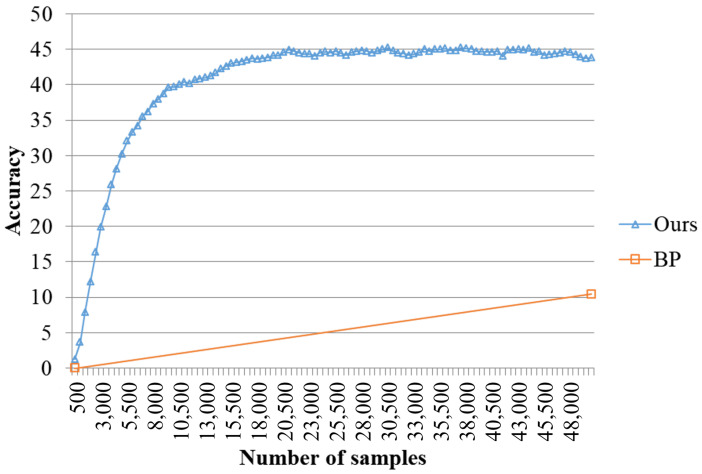
Random input comparison of the proposed model with one epoch backpropagation on Cifar-100.

Figure 5 shows the test result with the one epoch training. Our proposed model outperforms conventional backpropagation. The results show that the proposed incremental QR based learning is very efficient and powerful for datum-wise incremental online learning, whereas all of the recent incremental learning methods are based on backpropagation. The final accuracy of the proposed model is 43.8 and the maximum accuracy is 45.25, but the accuracy by one epoch backpropagation learning is 10.43.

### 4.3. Comparison with Replay Memory-Based Methods

There is a very recent paper, ER-MIR [23], that considers the online concept in continual learning. The major difference with our model is they use replay memory. We compared GEM [40], iCarl [5], and ER-MIR [23] with our proposed model on Cifar-10. For the paired comparison, the proposed model is trained at the same setting condition as shown in Section 4.1.

Table 1 shows that our proposed method without a replay memory to keep old data outperforms conventional replay memory-based incremental learning algorithms for both accuracy and forgetting. Note that M indicates memory size per class.

### 4.4. Computational Efficiency of the Proposed Incremental QR Factorization Compared with That of Batch

**Figure 6:**
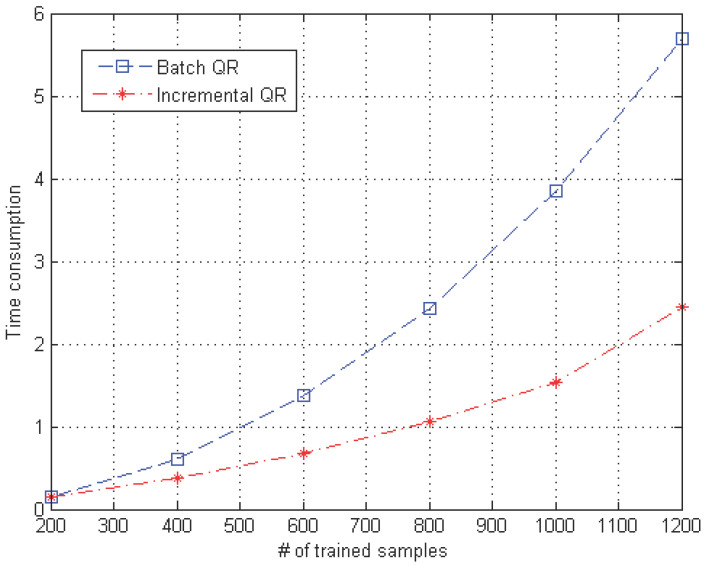
Number of training samples versus time consumption for the batch and incremental QR methods. (Incremental QR consumes less time than batch QR as the number of training samples increases).

Figure 6 shows that the incremental QR is almost twice as fast as the batch-updating method. This fast learning time results from the efficient updating method used by the proposed incremental QR compared to batch learning, which computes the entire process repeatedly when new data are given. In particular, as the data size increases, the time efficiency of the proposed incremental QR is considerably improved.

### 4.5. Hyper-Parameter Effect Analysis

We applied hyper-parameter change analysis on the random incremental experiment setting of the proposed method.

#### 4.5.1. Small ϵ

**Figure 7:**
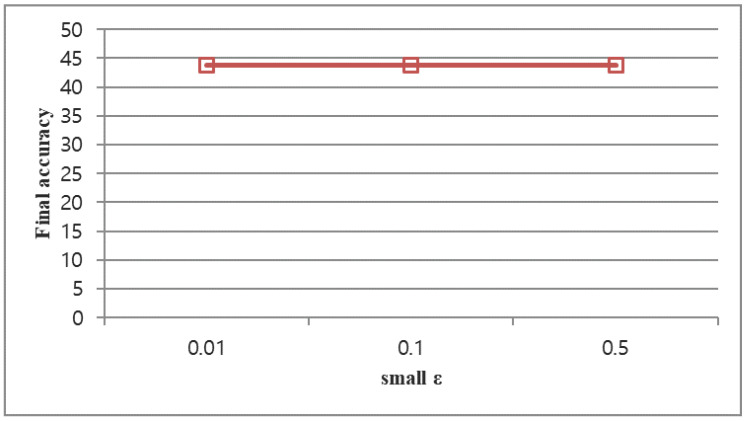
Random incremental test final accuracy change corresponds to small ϵ changes.

As we can see in Figure 7, a small change of ϵ does not significantly affect the final result. Therefore, our selection of ϵ = 0.1 in the paper is acceptable.

#### 4.5.2. Bias Selection Parameter r

**Figure 8:**
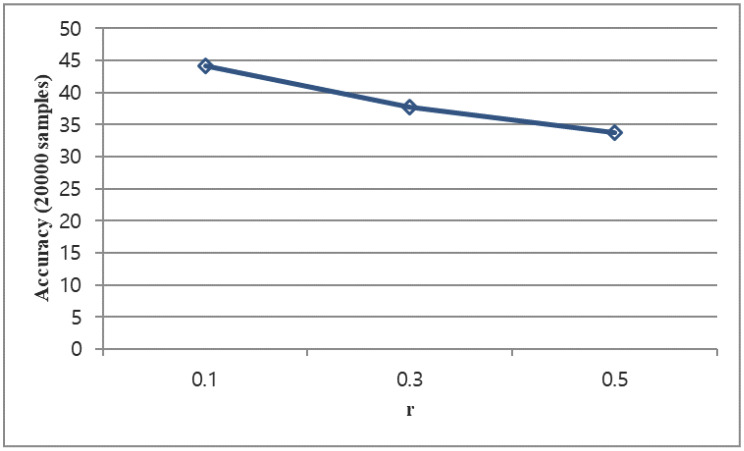
Random incremental test final accuracy change corresponds to bias selection parameter r changes.

We changed r from 0.1 to 0.5 in 0.2 units. The case of r = 0.1 is most effective. Figure 8 indicates that as the effect of bias increases by increasing the r, the accuracy decreases. Therefore, we see that the selection of r = 0.1 is correct.

## 5. Conclusions and Future Work

We propose a novel datum-wise online incremental learning algorithm that adopts effective maxima and boundary concepts to find incremental weight magnitude and bias. Our method adopts an incremental QR factorization algorithm to find incremental weight shapes. By combining those new concepts in conventional deep neural networks, we can find an appropriate weight and bias for the datum-wise online incremental learning. Our experimental results show that the proposed method outperforms conventional backpropagation-based class-wise incremental learning methods. Current and recent lifelong learning approaches assume the situation that the training data is already well prepared for each different task. However, practical real-world applications are tougher than the lab environment. Tasks such as face recognition, traffic surveillance, and even any object recognition tasks, will have new persons, brand-new cars, and newly invented objects. The major scope of our proposed model is to deal with these kinds of problems. The limitation of our work is that it is hard to fairly compare the performances with the class-wise incremental learning approaches. Since the proposed method is datum-wise, the absolute performance cannot be superior to those recent class-wise approaches. In future work, we will consider the mini-batch problem to meet practical necessities and also find a way to update hidden representations in a similar way.

## Figures and Tables

**Table 1:** Comparison with replay memory-based methods on Cifar-10. The bold numbers are the best results.

	Accuracy			Forgetting		
**Methods**	**M = 20**	**M = 50**	**M = 100**	**M = 20**	**M = 50**	**M = 100**
GEM [40]	16.8	17.1	17.5	73.5	70.7	71.7
iCarl [5] (5 iter)	28.6	33.7	32.4	49	40.6	40
ER-MIR [23]	29.8	40.0	47.6	50.2	**30.2**	**17.4**
DOI (Ours)	**50.4** (M = 0)			**48.9** (M = 0)		

## Data Availability

This study uses the following publicly available datasets: CIFAR-10 and CIFAR-100. These data can be found here: https://www.cs.toronto.edu/~kriz/cifar.html (accessed on 7 August 2023).
